# QuickStats

**Published:** 2015-04-17

**Authors:** FROM THE NATIONAL CENTER FOR HEALTH STATISTICS

**Figure:**
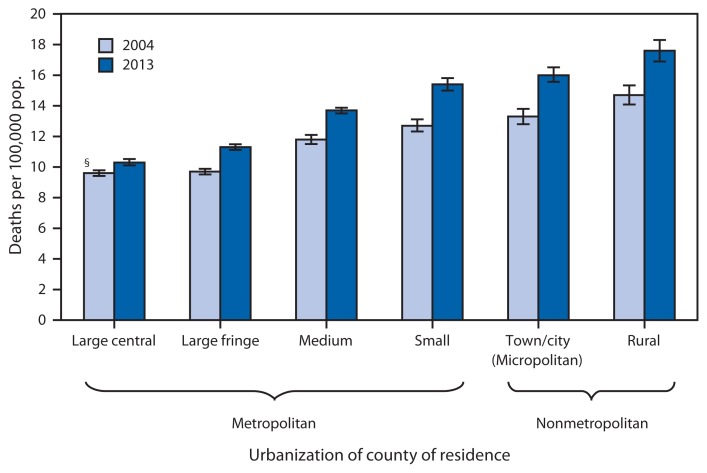
Age-Adjusted Rates for Suicide,* by Urbanization of County of Residence^†^ — United States, 2004 and 2013 * Age-adjusted rates per 100,000, based on the 2000 U.S. standard population. Suicides are coded as *U03, X60–X84, and Y87.0 in the *International Classification of Diseases, 10th Revision*. ^†^ Counties were classified into urbanization levels based on a classification scheme that considers metropolitan/nonmetropolitan status, population, and other factors. ^§^ 95% confidence interval.

The overall age-adjusted suicide rate was 11.0 deaths per 100,000 population in the United States in 2004 and 12.6 in 2013. From 2004 to 2013, the suicide rate increased in all county urbanization categories, with the smallest increase (7%) in large central metropolitan counties and the largest increases in small metropolitan, town/city (micropolitan) and rural counties (approximately 20% in each). For both years, suicide rates were increasingly higher as counties became less urbanized. For 2013, the age-adjusted suicide rate in rural counties was 1.7 times the rate for large central metropolitan counties (17.6 compared with 10.3 deaths per 100,000).

**Sources:** National Vital Statistics System. County-level mortality file. Available at http://wonder.cdc.gov/mortSQL.html. Ingram DD, Franco SJ. 2013 NCHS urban-rural classification scheme for counties. Vital Health Stat 2014;2(166). Available at http://www.cdc.gov/nchs/data/series/sr_02/sr02_166.pdf.

**Reported by:** Li-Hui Chen, PhD, eyx5@cdc.gov, 301-458-4446; Deborah D. Ingram, PhD.

